# High-dose linezolid during BPaL implementation for pre-extensively drug-resistant TB or failure/intolerance of rifampicin-resistant TB regimens

**DOI:** 10.5588/ijtldopen.26.0105

**Published:** 2026-07-13

**Authors:** T.M.P. Nguyen, T.T.H. Nguyen, T.H. Nguyen, F. Wares, M. Mbenga, A. Gebhard, V.L. Dinh, B.H. Nguyen, T.T.T. Hoang, V.N. Nguyen, S. Callens, T. Decroo

**Affiliations:** 1 National Lung hospital, Hanoi, Vietnam;; 2 Department of Internal Medicine & Infectious Diseases, Ghent University Hospital, Ghent, Belgium;; 3 Institute of Tropical Medicine Antwerp, Antwerp, Belgium;; 4 KNCV, Hanoi, Vietnam;; 5 KNCV, the Hague, the Netherlands;; 6 Hanoi Medical University, Hanoi, Vietnam;; 7 Vietnam National University Ha Noi, University of Medicine and Pharmacy, Hanoi, Vietnam.

**Keywords:** tuberculosis, drug-resistant TB, high drug dosage, BPaL regimen

Dear Editor,

Vietnam has a high burden of TB and rifampicin-resistant TB (RR-TB).^1^ The prevalence of additional resistance to fluoroquinolones (FQ), or pre-extensively drug-resistant TB (pre-XDR-TB), among RR-TB is approximately 17.9%.^2^ Pre-XDR-TB used to be treated with long (18–24 months) individualised regimens, resulting in poor outcomes.^3^ Following the Nix trial, a 6–9-month regimen comprising bedaquiline (BDQ/B), pretomanid (Pa), and linezolid (LZD/L) – BPaL regimen – was recommended for patients with pre-XDR-TB, RR-TB treatment failure, or intolerance to RR-TB regimen.^4,5^ In the Nix trial, a high-dose of LZD (1,200 mg) was used. In Vietnam, the same high-dose LZD regimen (BPaL^h^) was evaluated through a prospective observational cohort study, to assess the occurrence of adverse events (AE) and end-of-treatment outcomes. Although the recommended dose of LZD in the BPaL regimen was reduced after the ZeNix trial (from 1,200 mg to 600 mg daily),^6,7^ our comprehensive assessment of BPaL^h^ in Vietnam adds valuable insights. To the best of our knowledge, this is the first study showing safety data on BPaL^h^ from a non-trial setting. Two BPaL studies conducted in South-East Asia (Indonesia and Thailand) reported the outcomes of the regimen with LZD at 600 mg or 1,200 mg daily, but without detailed safety analysis.^8,9^ LZD toxicity differs from region to region^10^ and neither the Nix or ZeNix trial included patients from South-East Asia.^4,6^

Seventy-three patients were treated with BPaL^h^ for 6–9 months at four treatment centres in Vietnam from November 2021 to December 2023. RR-TB was diagnosed with Xpert MTB/RIF (Cepheid Inc., USA), followed by FQ resistance screening using genotypic (GenoType MTBDRsl, Hain Lifescience, Germany) or phenotypic second-line drug susceptibility testing. Inclusion criteria for BPaL^h^ comprised having 1) bacteriologically confirmed pre-XDR-TB or 2) intolerance or failure of non-BDQ RR-TB regimens. Exclusion criteria were severe allergy, confirmed resistance, or previous exposure (2 weeks or longer) to any of the BPaL drugs; severe extra-pulmonary TB; and pregnancy or breastfeeding. Eligible patients received treatment for 26 weeks, with extension to 39 weeks if delayed culture conversion or lack of clinical response at month 4. AE management adhered to Vietnam’s active drug safety monitoring (aDSM) framework (intermediate package). AEs of special interest (AESI) for BPaL^h^ included myelosuppression (MS), peripheral neuropathy (PN), optic neuritis, QT prolongation, and hepatoxicity (HP). AE were graded using the NTP’s aDSM grading scale.

Of the 73 patients, 39 (53%) were male. The median age was 38.5 years (interquartile range: 28–52), and the mean body mass index (±sd) was 19.2 (±2.5). Three (4.1%) were HIV-positive, all on antiretroviral therapy prior to BPaL^h^ initiation. Most patients (68; 93%) had pre-XDR-TB, while 5 (7%) were treated after RR-TB treatment failure or intolerance. At the end of treatment, 93% achieved treatment success: 86% were cured and 7% completed treatment. None had treatment failure, 1 patient (1%) died and 4 (6%) were lost to follow-up. Thirty (41.1%) experienced at least one AE during BPaL^h^. Twenty patients (27%) had Grade 3–4 AEs. The patient who died was without recorded AESI. Figure shows AEs, their management, and outcomes. MS (16/30; 53%) and PN (11/30; 37%) were most common. One patient experienced HP, one had Grade 2 rash, and one had an AE of unknown type. Among 30 with AEs, LZD was interrupted in 9 (30%) and stopped in 13 (43%). The entire regimen was interrupted in 3 (10%).

**Figure.**
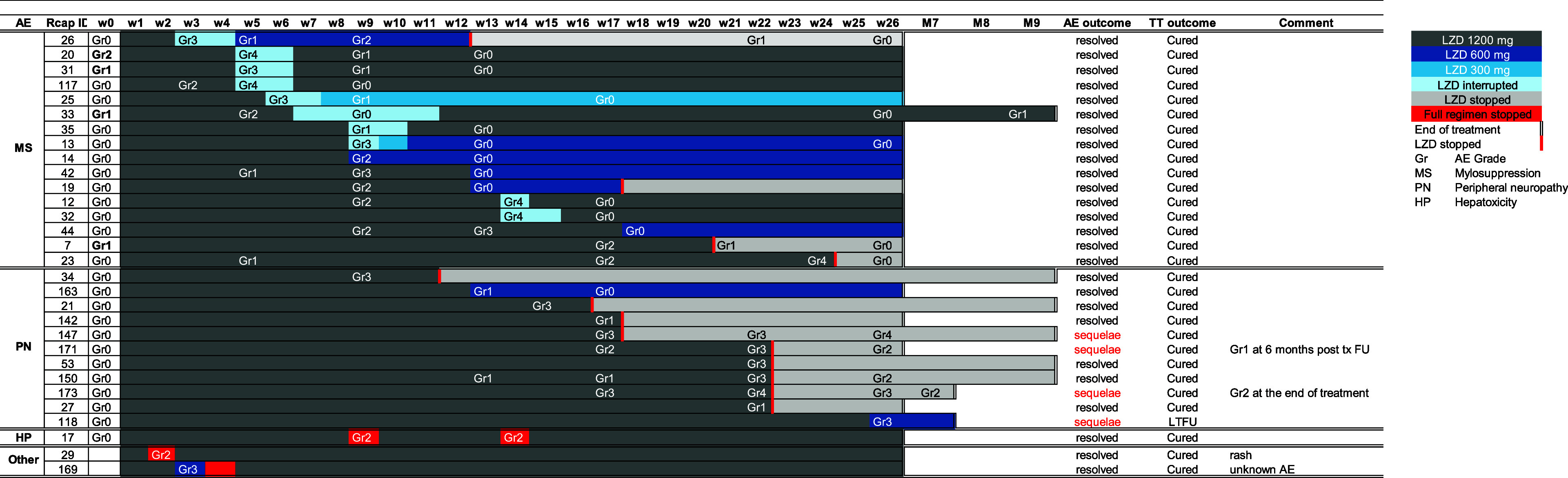
Treatment duration, linezolid (LZD) dose reductions, LZD interruptions, and outcomes among 30 patients with adverse events (AEs) during treatment with BPaL^h^ in Vietnam. w0 = week 0; M7 = month 7; AE outcome = outcome of adverse event; TT outcome = treatment outcome; Gr0 = AE grade 0; MS = myelosuppression; PN = peripheral neuropathy; HP = hepatoxicity; post tx FU = post-treatment follow-up.

Among 16 patients with MS, the onset typically occurred within the first two treatment months. Severity varied; 10 had Grade 3–4 MS. Management strategies included temporary LZD interruption followed by dose reduction or reintroduction at the original dose, depending on the evolution. Of the 16 patients, 4 required permanent LZD cessation (2 after dose reduction, 2 immediately), and 9 patients had LZD temporarily interrupted for 1–2 weeks, then restarted at either 300 mg (1 patient), 600 mg (2 patients), or at 1,200 mg (7 patients). The other three patients saw resolution of MS with LZD dose reduction to 600 mg, without treatment interruption. The majority (15/16) of LZD interruptions or dose reductions did not result in treatment prolongation. Only one required extending to 39 weeks. While MS was frequent, resulting in early temporary drug interruptions and dose reductions, this management proved effective, without impacting treatment outcomes. Because of this early onset, clinical guidelines recommend weekly monitoring of full blood counts for at least the first 2–3 months of treatment. A haemoglobin drop of more than 10% at 4-week mark may strongly predict subsequent severe anaemia.^11^

Among 11 patients with PN, initial symptoms were identified mostly from month 3 onwards and graded by the Brief Peripheral Neuropathy Screen or patients’ symptoms. In most cases (9/11; 12% of 73), LZD was permanently discontinued. Over half of these patients (5/9) required treatment prolongation. The other two patients had LZD reduced to 600 mg. While most PN cases were resolved (downgrade of AEs and no complaint of symptoms at the patient’s last visit), three patients experienced ongoing sequelae at the completion of treatment. One patient with Grade 3 PN was lost to follow-up. Also in Nix-TB, with 1,200 mg daily for 26 weeks, PN required treatment cessation in 15% of patients, while treatment was often prolonged.^4^ This finding emphasises the necessity of close neurological monitoring and timely LZD dose adaptations. Structured dose reduction, to 300 mg daily in BPaL regimens, could ensure effectiveness with fewer cases of peripheral neuropathy, as endorsed by the WHO.^7,12^

Only one patient experienced HP (3.3% of those with AEs), requiring two separate 1-week treatment interruptions due to Grade 2 liver enzyme elevations. The patient was able to complete the BPaL^h^ regimen after 26 weeks. Although less frequent, HP requires close monitoring and can be managed if detected and addressed promptly. The patient with the rash and the patient with the AE of unknown origin both required a 1-week treatment interruption, after which the AEs resolved, and both patients successfully completed the full BPaL^h^ regimen.

In our study, the incidence of AEs was high. The 27% Grade 3–4 AE rate was slightly less than 31%–35% in the 1,200 mg LZD-throughout arms in Nix and ZeNix, but higher than the 20% in the 600 mg LZD-throughout arm of ZeNix.^4,6^ Despite the high AE rate, our treatment success rate remains impressive (93%). In other BPaL^h^ cohorts, treatment success was quite similar: 92% in Nix-TB,^4^ 93% in ZeNix-TB, and 98% in Indonesia’s cohort (with 63 out of 84 patients with 1,200 mg LZD).^6,9^ Indeed, BPaL^h^ regimen is highly effective in treating drug-resistant TB. In our study, post-treatment follow-up is ongoing to assess relapses, especially in patients who had LZD permanently stopped.

This prospective study added evidence with BPaL^h^ from a programmatic setting. It showed that findings from previous trials on 1,200 mg LZD in BPaL also apply to the South-East Asian context. Limitations are the small sample size and observational design. Recent studies about structured dose reduction of LZD in BPaL based regimens,^12–14^ or alternative oxazolidinones with comparable efficacy and markedly reduced toxicity,^15^ provided promising evidence for an effective and safer 6–9-month regimens in pre-XDR/RR-TB treatment. Nevertheless, the role of AE management according to aDSM guidelines remains essential to ensure patient safety.
